# Global status and trends in heart failure with preserved ejection fraction over the period 2009-2020: A bibliometric analysis

**DOI:** 10.1097/MD.0000000000029106

**Published:** 2022-03-18

**Authors:** Qiuju Dong, Junwei Zhang, Qinghua Han, Hongzhen Zhang, Meiling Wang, Qi Huang, Jianping Zhao

**Affiliations:** ^a^ *Hospital of Integrated Traditional and Western Medicine, Shanxi University of Chinese Medicine, Taiyuan, Shanxi, China,* ^b^ *Third Clinical College, Shanxi University of Chinese Medicine, Taiyuan, Shanxi, China,* ^c^ *The Key Research Laboratory of Benefiting Qi for Acting Blood Circulation Method to Treat Multiple Sclerosis of State Administration of Traditional Chinese Medicine, Research Center of Neurobiology, Shanxi University of Chinese Medicine, Jinzhong, Shanxi, China,* ^d^ *School of Management, Shanxi Medical University, Jinzhong, Shanxi, China,* ^e^ *School of Computer and Information Technology, Shanxi University, Taiyuan, Shanxi, China,* ^f^ *First Hospital, Shanxi Medical University, Taiyuan, Shanxi, China.*

**Keywords:** bibliometric analysis, heart failure, heart failure with preserved ejection fraction, research status, research trends

## Abstract

**Background:**

Heart failure with preserved ejection fraction (HFpEF) comprises about 50% of the cases of heart failure (HF), but so far there is no effective treatment strategy. This study used bibliometric methods to analyze the scientific literature on HFpEF in 2009 to 2020, and evaluate the global scientific output of HFpEF research, in order to explore the research status and trends in this field.

**Methods:**

Documents about the HFpEF research published in 2009 to 2020 were retrieved from Science Citation Index Expanded (SCIE) in Web of Science. This study used bibliometrix R-package, VOSviewer, and CiteSpace to conduct the bibliometric analysis.

**Results:**

A total of 1971 documents (1508 articles and 283 reviews) were retrieved to construct the local HFpEF literature collection for analysis. The number of annual documents had increased year by year in general, from 24 to 353. Relevant documents were mainly written in English, and mostly focused on the field of “Cardiovascular System Cardiology.” USA ranked first in the relevant countries/regions with most documents, and the leading affiliation was Mayo Clin. Shah SJ was the most productive author, while Borlaug BA ranked highest among the local cited authors and G-index. Circulation was the most local cited source, while Eur J Heart Fail published the most documents and was rated as the top source in terms of G-index. “Paulus WJ, 2013, J Am Coll Cardiol” was the top local cited document within the local HFpEF literature collection, while “Owan TE, 2006, New Engl J Med” outside the local HFpEF literature collection was the most local cited reference. The keywords such as “mortality,” “dysfunction,” “diagnosis,” “outcomes,” and “diastolic dysfunction” were most frequent, while “hemodynamics,” “comorbidity,” “myocardial infarction,” “inflammation,” and “phenotype” indicated research frontiers or emerging trends. Furthermore, this study also found some deeper bibliometric relationships through bibliographic networks.

**Conclusions:**

Due to the multi-dimensional bibliometric analysis, this study shows a wide view of scientific productivity related to HFpEF, and provides valuable guidance for researchers interested in HFpEF, assisting them in understanding the research status, identifying potential collaborators, discovering research hotspots and frontiers, and conducting more in-depth research.

## 1. Introduction

Heart failure (HF) is the terminal stage of various cardiac diseases, with significant morbidity and high mortality worldwide.^[2]^ Heart failure with preserved ejection fraction (HFpEF) is the most common type of HF, and it accounts for more than half of the cases of HF.^[1,4-14]^ HFpEF is characterized by normal or near normal left ventricular ejection fraction,^[15]^ so the prognosis of HFpEF patients is often falsely considered to be better than that of heart failure with reduced ejection fraction (HFrEF) patients.^[3]^ However, the morbidity, mortality, acute exacerbation rate, readmission rate, and prognosis of HFpEF are all similar to those of HFrEF.^[1,3,11,13-17]^

In fact, the etiology, pathophysiology, and management of HFpEF and HFrEF are very different.^[1,18]^ In the past 2 decades, the standard treatment of HF has been popularized, which has significantly reduced the hospitalization rate and improved the survival rate of patients with HFrEF.^[1]^ HFpEF currently lacks a successful treatment strategy to improve prognosis. This is attributed to incomplete understanding of HFpEF pathophysiology, patient heterogeneity, and mismatch between treatment mechanisms and major pathophysiologic processes.^[19]^ HFpEF is associated with a variety of medical complications and pathophysiological pathways or clinical phenotypes, accompanied by multiple organ system disorders.^[1,12,24]^ The important clinical phenotypes of HFpEF include diabetes, hypertension, lung disease, chronic kidney disease, obesity, and so on.^[12]^ Personalized techniques based on individuals’ phenotypic traits, diagnosis, and pathophysiology stratification are needed to replace “one-size-fits-all” therapy for HFpEF.^[19,22,25]^ HFpEF, in particular, should be handled differently depending on its phenotypes.^[3,19]^ Only addressing phenotypic diversity of HFpEF can tailor more personalized therapies for specific HFpEF phenotypes.^[20]^ In summary, HFpEF represents one of the largest unmet clinical needs in the current cardiovascular medicine,^[2,25-27]^ so there is an urgent need to develop new treatments for patients with HFpEF.^[1]^

Clinical researchers should extensively collect literature and accumulate relevant professional knowledge in the direction of related disciplines to ensure the innovation, scientificity, and non-repetitiveness of the clinical research topics. Bibliometrics is a widely accepted research method, which has been carried out in clinical research topics. Based on statistics and visualization technology, bibliometrics makes quantitative and qualitative analysis on the published academic literature in a specific clinical research topic, describes knowledge structure and tracks development trend. Bibliometric analysis usually includes the following steps: putting forward a research question, retrieving databases, collecting and analyzing data, drawing maps, preparing, and publishing the report.^[28]^ Some bibliometric tools have been widely used by researchers to evaluate the impacts of documents, contributions of authors/affiliations/countries, patterns of authorship, and the main directions of future research in a clinical research topic. Clinical researchers should develop search strategies, retrieve relevant documents from literature databases, and use bibliometric methods to conduct multifaceted bibliometric analysis of these documents to find clinical research hotspots and determine research directions in a purposeful and targeted manner. At present, there are some bibliometric studies in the field of cardiovascular diseases,^[29-32,45]^ one of which is on HFpEF. A bibliometric study conducted by Edlinger et al^[45]^ showed a great increase in research on HFpEF in the United States, Japan, and continental Europe, and identified RAAS-blockade and 5-phosphodiesterase-inhibition as a therapeutic trend in HFpEF. This study will take the bibliometric analysis of HFpEF a step further to provide a more comprehensive perspective for clinical researchers.

## 2. Methods

This study retrieved literature collection from Web of Science. Web of Science (WoS) is one of the world’s most trusted publisher-independent global citation databases, and it allows users to explore the literature of various scientific fields. The literature retrieval details in this study are as follows:

Data Source: Web of Science.

Database: Web of Science Core Collection.

Edition: Science Citation Index Expanded (SCIE).

Search Option: Advanced Search.

Query: TI = (HFpEF Or heart failure with preserved ejection fraction) and PY = (2009-2020) and DT = (Article Or Review).

Record Content: full record and cited references.

Data Format: Plaintext.

Query Data: July 7, 2021.

In the query, TI, PY, and DT stand for title, year published, and document type respectively. In order to improve the accuracy of literature retrieval, this study used search terms “HFpEF” or “heart failure with preserved ejection fraction” in the title to retrieve articles or reviews between 2009 and 2020.

Most of data analyses in this study were carried out with bibliometrix R-package (version 3.1), VOSviewer (version 1.6.17), and CiteSpace (version 5.8.R1).^[33-35]^ These bibliometrics tools can process the HFpEF literature collection, carry out various quantitative analyses, and build matrices for author collaboration, bibliographic coupling, references co-citation, and keyword co-occurrence networks.

## 3. Results

### 3.1. Descriptive analysis

This study used WoS Results Analysis Tool and bibliometrix R-package to perform a descriptive analysis of the local HFpEF literature collection. In Table 1, some statistics information are reported.

**Table 1:** Some statistical information about the HFpEF local literature collection.

**Description**		**Results**
Sources		326
Publications		1791
	Articles	1508
	Reviews	283
References		30,544
All Keywords		4127
	Author Keywords (DE)	1998
	Keywords Plus (ID)	2565
Authors		7712
Author Appearances		15,569
HFpEF = heart failure with preserved ejection fraction.		

The local HFpEF literature collection contained 1791 documents from 326 sources, including 1508 articles and 283 reviews. In these documents, a total of 7712 authors appeared 15,569 times and a total of 30,544 references were cited. WoS employs 2 types of keywords.

Author Keywords (DE), listed by the original authors, and Keywords Plus (ID), annotated by an automatic computer algorithm. The number of all keywords including Author Keywords and Keywords Plus was 4127. Author Keywords and Keyword Plus had 1998 terms and 2565 terms respectively, and there were duplicates between them.

Figure 1 shows the annual distribution of 1791 documents between 2009 and 2020. Except that the number of documents in 2019 decreased slightly compared with that in 2018, the number of documents showed an overall upward trend over year, increasing from 24 in 2009 to 353 in 2020, with an average annual growth rate of about 27.69%.

**Figure:**
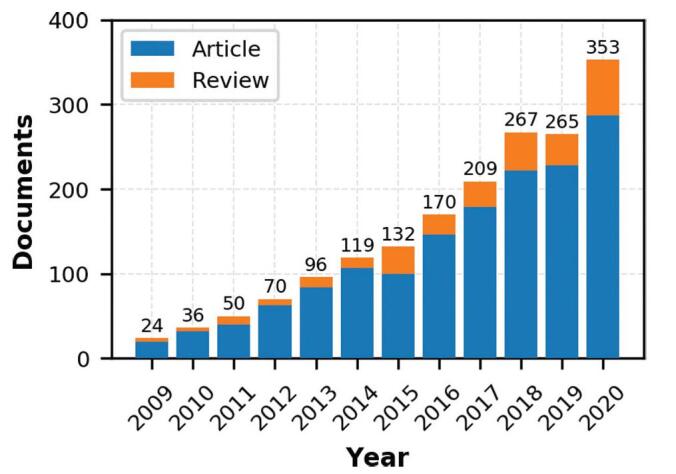
**Figure 1.** Annual scientific production.

Figure 2 in which all the data were from WoS Results Analysis Tool, provides some snapshots of the top-10 relevant languages, research areas, countries/regions, and affiliations.

**Figure:**
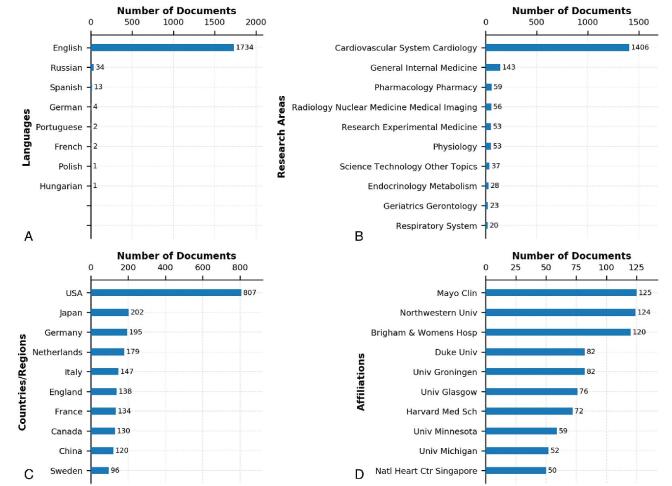
**Figure 2.** Some snapshots about HFpEF research. (A) Relevant language. (B) Most relevant research areas. (C) Most relevant countries/regions. (D) Most relevant affiliations. HFpEF = heart failure with preserved ejection fraction.

Figure 2A shows the relevant languages. English was the language most used by authors to write HFpEF documents. There were 1734 English documents, accounting for the vast majority of all documents. The other 2 languages used more were Russian and Spanish, with 34 and 13 documents respectively. In addition, a few documents were written in German, Portuguese, French, Polish, and Hungarian.

HFpEF documents in the local HFpEF literature collection covered 43 research areas. Figure 2B shows top-10 most relevant research areas. “Cardiovascular System Cardiology” was the leading research area having 1406 documents, followed by “General Internal Medicine,” “Pharmacology Pharmacy,” and “Radiology Nuclear Medicine Medical Imaging” with 143, 59, and 56 documents respectively.

A total of 79 countries/regions contributed to the global HFpEF document output. Figure 2C shows the top-10 most relevant countries/regions. Most were developed countries/regions, of which USA ranked first with 807 documents, followed by Japan, Germany, and Netherlands with 202, 195, and 179 documents respectively. As the only developing country/region in the top-10, China had produced 120 documents, ranking ninth.

The 1791 documents were published by 1983 research affiliations. Figure 2D shows top-10 most relevant affiliations. Mayo Clin was the most productive affiliation with a total of 125 documents, followed by Northwestern University and Brigham & Womens Hospital with 124 and 120 documents respectively. Among the top-10 affiliations in the global HFpEF document output, only 3 were not in USA.

### 3.2. Author and source analysis

Authors and sources are important research constituents in bibliometrics. Figure 3 in which all the data were from R bibliometrix, used different criteria to rank authors and sources in the local HFpEF literature collection.

**Figure:**
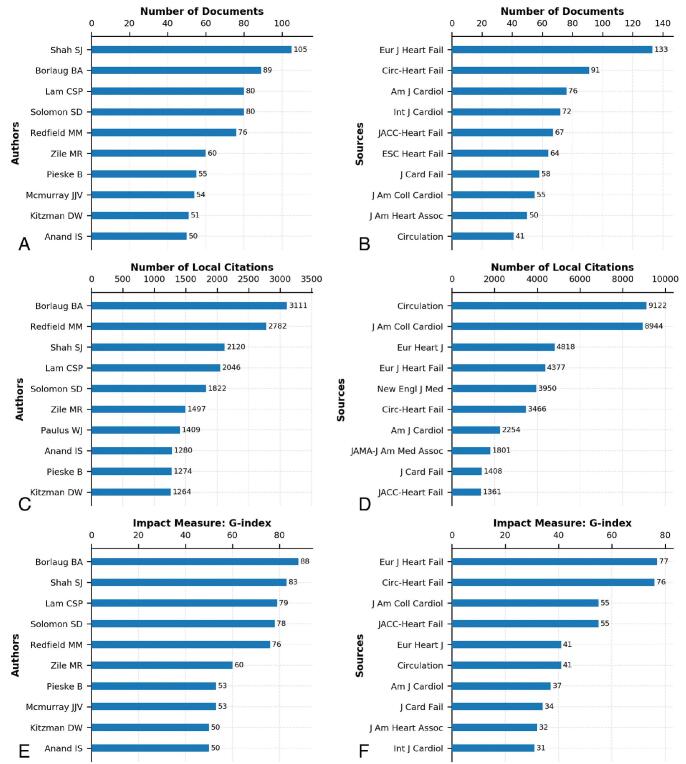
**Figure 3.** Rankings of authors and sources. (A) Most relevant authors. (B) Most relevant sources. (C) Most local cited authors. (D) Most local cited sources. (E) Author local impact by G-index. (F) Source local impact by G-index.

The top-10 authors in terms of the total numbers of documents are listed in Fig. 3A. All authors were having ≥50 documents, while Shah SJ ranked first, publishing 105 documents. Borlaug BA, Lam CSP, and Solomon SD were other major contributing authors with 89, 80, and 80 documents respectively. The most relevant authors ranking 5 to 10 were Redfield MM, Zile MR, Pieske B, Mcmurray JJV, Kitzman DW, and Anand IS.

The top-10 most productive sources are presented in Fig. 3B. They published a total of 707 documents which accounted for 39.5% of all documents included in the local HFpEF literature collection. The source published the most HFpEF documents was Eur J Heart Fail, with 133 documents, followed by Circ-Heart Fail, Am J Cardiol, and Int J Cardiol with 91, 76, and 72 documents respectively. JACC-Heart Fail, ESC Heart Fail, J Card Fail, J Am Coll Cardiol, J Am Heart Assoc, and Circulation were also sources that were clearly of interest to HFpEF research.

This paper introduced two concepts: global citation and local citation. Global citations means the citations that a local document has received from other documents indexed on WoS, while local citations means the citations that a reference, included in the bibliographies of the local documents, has received from the local documents. The diagram of global citations and local citations is shown in Fig. 4. In this paper, the concept of global citation is applicable to a local document, while the concept of local citation is applicable to a reference cited by the local documents. If a document has a high number of local citations, it means that it is an important document in the field. A local cited document is a local document cited by other local documents, so it belongs both in the document set and in the reference set.

**Figure:**
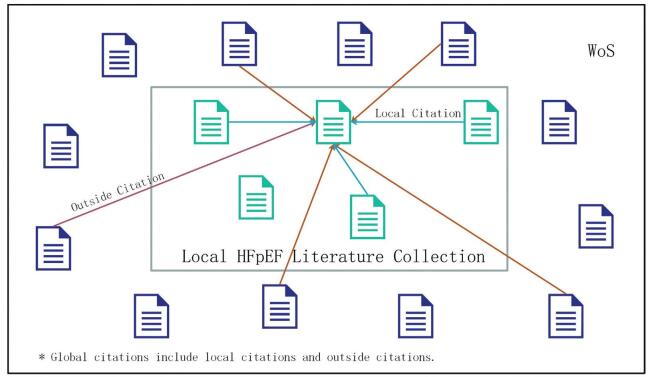
**Figure 4.** Diagram of global citations and local citations.

This paper lists the top-10 authors according to their local citations in Fig. 3C. The top-3 most local cited authors were as follows: Borlaug BA (3111 local citations), Redfield MM (2782 local citations), Shah SJ (2120 local citations).

Figure 3D lists the top-10 sources in terms of their local citations. Circulation topped the list with 9122 local citations. J Am Coll Cardiol ranked second, with a total of 8944 local citations. Eur Heart J was cited 4818 times locally, ranking third.

Author impact metrics can be used to evaluate the impact of the academic documents of an author or a source, and they are usually based on the number of citations and the number of documents. The commonly used author impact metrics include H-index, G-index, and M-index. H-index is the most used author impact metric, while G-index that gives more weight to highlycited document is an alternative to H-index.^[36]^ This study adopted G-index to measure the local impact of authors and sources in the collection. The author local impact and source local impact by G-index are shown in Fig. 3E and F.

As shown in Fig. 3E, all authors showed G-index scores of ≥50, of which Borlaug BA had the highest G-index score of 88, followed by Shah SJ and Lam CSP. As shown in Fig. 3F, Eur J Heart Fail was the source with the highest G-index score of 77, and Circ-Heart Fail followed, with a G-index score of 76, one step away from the top of the list. All authors and sources in Fig. 3 played important roles on HFpEF research.

The top-10 authors with most local impact were identified by G-index. Figure 5 uses the timeline to show these top authors’ production over the years. The production of an author referred to the number of published documents and the global citations of these documents. For example, Borlaug BA published 2 documents in 2009, which were cited 910 times in WoS since 2009. From Fig. 5, the research dynamics of these top authors can be observed.

**Figure:**
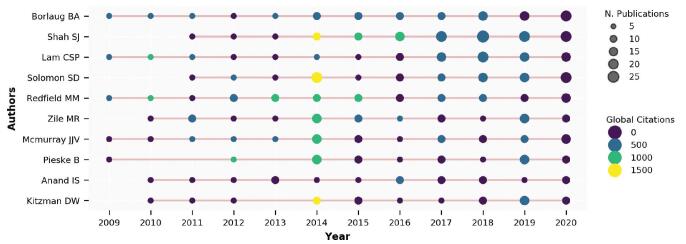
**Figure 5.** Top-authors’ production over the years.

### 3.3. Document and reference analysis

As listed in Table 2, top-20 most local cited documents included 17 articles and 3 reviews, of which “Paulus WJ, 2013, J Am Coll Cardiol” ranked first. All 20 documents were published before 2018. Further observation found that most of these documents were published between 2011 and 2013. This phenomenon indicates that the research foundation of HFpEF was basically formed between 2011 and 2013. Circulation followed by J Am Coll Cardiol were major contributors to these most local cited documents. Figure 6 uses the historical direct citation network to reveal the reference relationship between these top-20 most local cited documents.

**Table 2:** Top-20 most local cited documents.

**Rank**	**Title**	**Document information**	**Type**	LC*	**GC**†
1 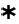	A Novel Paradigm for Heart Failure With Preserved Ejection Fraction: Comorbidities Drive Myocardial Dysfunction and Remodeling Through Coronary Microvascular Endothelial Inflammation	Paulus WJ, 2013, J Am Coll Cardiol, V62, P263	Review	440	1472
2 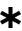	Spironolactone for Heart Failure With Preserved Ejection Fraction	Pitt B, 2014, New Engl J Med, V370, P1383	Article	337	1099
3 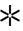	Effect of Phosphodiesterase-5 Inhibition on Exercise Capacity and Clinical Status in Heart Failure With Preserved Ejection Fraction: A Randomized Clinical Trial	Redfield MM, 2013, JAMA-J Am Med Assoc, V309, P1268	Article	238	705
4 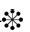	Heart Failure With Preserved Ejection Fraction: Pathophysiology, Diagnosis, and Treatment	Borlaug BA, 2011, Eur Heart J, V32, P670	Review	203	620
5 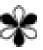	Exercise Hemodynamics Enhance Diagnosis of Early Heart Failure With Preserved Ejection Fraction	Borlaug BA, 2010, Circ-Heart Fail, V3, P588	Article	196	578
6 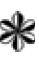	Pulmonary Hypertension in Heart Failure With Preserved Ejection Fraction: A Community-Based Study	Lam CSP, 2009, J Am Coll Cardiol, V53, P1119	Article	191	638
7 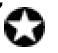	Global Cardiovascular Reserve Dysfunction in Heart Failure With Preserved Ejection Fraction	Borlaug BA, 2010, J Am Coll Cardiol, V56, P845	Article	186	405
8 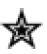	Trends in Patients Hospitalized With Heart Failure and Preserved Left Ventricular Ejection Fraction: Prevalence, Therapies, and Outcomes	Steinberg BA, 2012, Circulation, V126, P65	Article	173	444
9 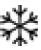	Phenotype-Specific Treatment of Heart Failure With Preserved Ejection Fraction: A Multiorgan Roadmap	Shah SJ, 2016, Circulation, V134, P73	Article	165	412
10	Effect of Spironolactone on Diastolic Function and Exercise Capacity in Patients With Heart Failure With Preserved Ejection Fraction: The Aldo-DHF Randomized Controlled Trial	Edelmann F, 2013, JAMA-J Am Med Assoc, V309, P781	Article	163	403
11	The Angiotensin Receptor Neprilysin Inhibitor LCZ696 in Heart Failure With Preserved Ejection Fraction: A Phase 2 Double-Blind Randomised Controlled Trial	Solomon SD, 2012, Lancet, V380, P1387	Article	160	635
12	Epidemiology and Clinical Course of Heart Failure With Preserved Ejection Fraction	Lam CSP, 2011, Eur J Heart Fail, V13, P18	Review	136	393
13	Impact of Noncardiac Comorbidities on Morbidity and Mortality in a Predominantly Male Population With Heart Failure and Preserved Versus Reduced Ejection Fraction	Ather S, 2012, J Am Coll Cardiol, V59, P998	Article	136	396
14	Coronary Microvascular Rarefaction and Myocardial Fibrosis in Heart Failure With Preserved Ejection Fraction	Mohammed SF, 2015, Circulation, V131, P550	Article	128	353
15	Relation of Disease Pathogenesis and Risk Factors to Heart Failure With Preserved or Reduced Ejection Fraction	Lee DS, 2009, Circulation, V119, P3070	Article	121	418
16	Phenomapping for Novel Classification of Heart Failure With Preserved Ejection Fraction	Shah SJ, 2015, Circulation, V131, P269	Article	116	408
17	Evidence Supporting the Existence of a Distinct Obese Phenotype of Heart Failure With Preserved Ejection Fraction	Obokata M, 2017, Circulation, V136, P6	Article	116	300
18	Exercise Training Improves Exercise Capacity and Diastolic Function in Patients With Heart Failure With Preserved Ejection Fraction Results of the Ex-DHF (Exercise training in Diastolic Heart Failure) Pilot Study	Edelmann F, 2011, J Am Coll Cardiol, V58, P1780	Article	107	358
19	The Survival of Patients With Heart Failure With Preserved or Reduced Left Ventricular Ejection Fraction: An Individual Patient Data Meta-Analysis	Berry C, 2012, Eur Heart J, V33, P1750	Article	106	425
20	Prevalence and Significance of Alterations in Cardiac Structure and Function in Patients With Heart Failure and a Preserved Ejection Fraction	Zile MR, 2011, Circulation, V124, P2491	Article	105	296

^*^LC: local citations within the local HFpEF literature collection.

^†^ GC: global citations in WoS.

**Figure:**
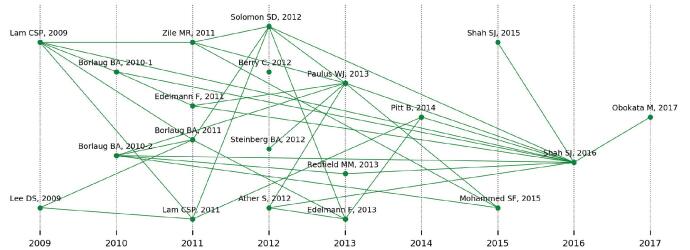
**Figure 6.** Historical direct citation network of top-20 most local cited documents.

A total of 30,544 references were cited by the local documents. Top-20 most local cited references are listed in Table 3, and “Owan TE, 2006, New Engl J Med” topped the list. Among the 20 references, 11 were published during 2009 to 2020, of which 9 were included in the local HFpEF literature collection, and the other 2 were guidelines for the diagnosis and treatment of heart failure. The 9 local references also appeared in Table 2, so there were duplicate items between the list in Table 3 and the list in Table 2. In order to compare the 2 lists, this paper added some special symbols to the duplicate items in Table 2, marked the corresponding special symbols in Note column in Table 3. New Engl J Med, J Am Coll Cardiol, Eur Heart J, and Circulation were the main sources of these most local cited references.

**Table 3:** Top-20 most local cited references.

**Rank**	**Title**	**Reference information**	**Type**	LC*	**Note**
1	Trends in Prevalence and Outcome of Heart Failure With Preserved Ejection Fraction	Owan TE, 2006, New Engl J Med, V355, P251	Article	660
2	016 ESC Guidelines for The Diagnosis and Treatment of Acute and Chronic Heart Failure	Ponikowski P, 2016, Eur Heart J, V37, P2129	Review	459
3	A Novel Paradigm for Heart Failure With Preserved Ejection Fraction: Comorbidities Drive Myocardial Dysfunction and Remodeling Through Coronary Microvascular Endothelial Inflammation	Paulus WJ, 2013, J Am Coll Cardiol, V62, P263	Review	440	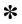
4	How to Diagnose Diastolic Heart Failure: A Consensus Statement on the Diagnosis of Heart Failure with Normal Left Ventricular Ejection Fraction by the Heart Failure and Echocardiography Associations of the European Society of Cardiology	Paulus WJ, 2007, Eur Heart J, V28, P2539	Article	383
5	Irbesartan in Patients with Heart Failure and Preserved Ejection Fraction	Massie BM, 2008, New Engl J Med, V359, P2456	Article	362
6	Effects of Candesartan in Patients with Chronic Heart Failure and Preserved Left-Ventricular Ejection Fraction: the CHARM-Preserved Trial	Yusuf S, 2003, Lancet, V362, P777	Article	360
7	Spironolactone for Heart Failure With Preserved Ejection Fraction	Pitt B, 2014, New Engl J Med, V370, P1383	Article	337	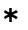
8	Outcome of Heart Failure with Preserved Ejection Fraction in a Population-Based Study	Bhatia RS, 2006, New Engl J Med, V355, P260	Article	333
9	The Perindopril in Elderly People With Chronic Heart Failure (PEP-CHF) Study	Cleland JGF, 2006, Eur Heart J, V27, P2338	Article	281
10	2013 ACCF/AHA Guideline for the Management of Heart Failure: A Report of The American College of Cardiology Foundation/American Heart Association Task Force on Practice Guidelines	Yancy CW, 2013, J Am Coll Cardiol, V62, Pe147	Article	253
11	Effect of Phosphodiesterase-5 Inhibition on Exercise Capacity and Clinical Status in Heart Failure With Preserved Ejection Fraction: A Randomized Clinical Trial	Redfield MM, 2013, JAMA-J Am Med Assoc, V309, P1268	Article	238	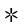
12	Heart Failure With Preserved Ejection Fraction: Pathophysiology, Diagnosis, and Treatment	Borlaug BA, 2011, Eur Heart J, V32, P670	Review	203	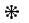
13	Exercise Hemodynamics Enhance Diagnosis of Early Heart Failure With Preserved Ejection Fraction	Borlaug BA, 2010, Circ-Heart Fail, V3, P588	Article	196	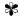
14	Pulmonary Hypertension in Heart Failure With Preserved Ejection Fraction: A Community-Based Study	Lam CSP, 2009, J Am Coll Cardiol, V53, P1119	Article	191	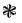
15	Global Cardiovascular Reserve Dysfunction in Heart Failure With Preserved Ejection Fraction	Borlaug BA, 2010, J Am Coll Cardiol, V56, P845	Article	186	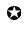
16	Diastolic Heart Failure — Abnormalities in Active Relaxation and Passive Stiffness of the Left Ventricle	Zile MR, 2004, New Engl J Med, V350, P1953	Article	181
17	Characteristics, Treatments, and Outcomes of Patients With Preserved Systolic Function Hospitalized for Heart Failure: A Report From the OPTIMIZE-HF Registry	Fonarow GC, 2007, J Am Coll Cardiol, V50, P768	Article	180
18	Impaired Chronotropic and Vasodilator Reserves Limit Exercise Capacity in Patients With Heart Failure and a Preserved Ejection Fraction	Borlaug BA, 2006, Circulation, V114, P2138	Article	174
19	Trends in Patients Hospitalized With Heart Failure and Preserved Left Ventricular Ejection Fraction: Prevalence, Therapies, and Outcomes	Steinberg BA, 2012, Circulation, V126, P65	Article	173	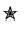
20	Phenotype-Specific Treatment of Heart Failure With Preserved Ejection Fraction: A Multiorgan Roadmap	Shah SJ, 2016, Circulation, V134, P73	Article	165	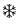

^*^ LC: local citations within the local HFpEF literature collection.

### 3.4. Keyword analysis

Keywords are terms that reflect the concept of a document topic and can provide a reasonable description of the research hotspot. This study integrated Author Keywords and Keywords Plus for overall keyword analysis. The local HFpEF literature collection contained 4127 keywords (including Author’s Keywords and Keywords Plus), and 3677 were left after manual processing of duplicate removal. The occurrences of 175 keywords reached or exceeded 20. After removing 6 keywords related to the search terms such as “heart failure,” “heart failure with preserved ejection fraction,” “preserved ejection fraction,” “HFpEF,” “left ventricular ejection fraction,” and “ejection fraction,” 169 keywords remained.

As shown in Fig. 7, “mortality,” “dysfunction,” “diagnosis,” “outcomes,” and “diastolic dysfunction” were the most frequent keywords, with the frequency of 386, 341, 340, 323, and 308 respectively.

**Figure:**
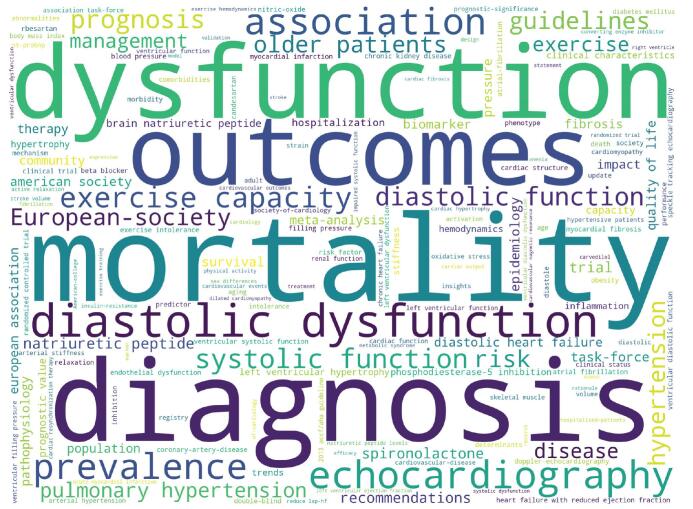
**Figure 7.** Keywords word cloud. The size of a keyword label is proportional to the number of occurrences of the keyword.

Burst keywords are regarded as indicators of research frontiers or emerging trends over time.^[30]^ In this study, CiteSpace was used for burst keyword detection. A total of 54 burst keywords were found, and 20 meaningful keywords were selected and listed in Table 4. Keywords such as “hemodynamics,” “comorbidity,” “myocardial infarction,” “inflammation,” and “phenotype” have emerged in recent years.

**Table 4:** Some burst keywords in the HFpEF research field.

**Keywords**	**Strength**	**Begin**	**End**	**2009-2020**
Doppler echocardiography	9.93	2009	2014	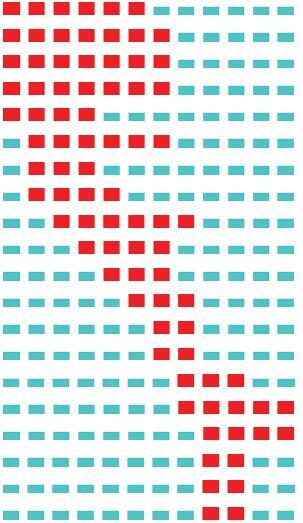
Brain natriuretic peptide	7.55	2009	2015	
Beta blocker	4.83	2009	2015	
Converting enzyme inhibitor	2.83	2009	2012	
Irbesartan	4.70	2010	2015	
Morbidity	3.91	2010	2012	
Blood pressure	3.28	2010	2013	
Risk factor	7.22	2011	2016	
Clinical characteristics	7.64	2012	2015	
Arterial stiffness	4.26	2013	2015	
Phosphodiesterase 5 inhibition	10.46	2014	2016	
Speckle tracking echocardiography	4.52	2015	2016	
Endothelial dysfunction	3.13	2015	2016	
Left ventricular hypertrophy	4.45	2016	2018	
Therapy	2.90	2016	2020	
Hemodynamics	10.43	2017	2020	
Comorbidity	5.80	2017	2018	
Myocardial infarction	4.23	2017	2018	
Inflammation	3.46	2017	2018	
Phenotype	11.19	2018	2020	
HFpEF = heart failure with preserved ejection fraction.				

### 3.5. Bibliographic networks

Bibliographic networks that include author collaboration network, bibliographic coupling network, references co-citation network, keyword co-occurrence network, and so on, can reveal the relationships between research constituents. This study used VOSviewer to perform bibliometric analysis and build networks.

Collaborations among authors have become more widespread as research methods and theories have become more complicated.^[37]^ Contributions from a variety of authors can provide deeper insights, and their collaboration can raise the study level. Author cooperation networks can disclose regular study groups, hidden academic teams, and core authors by displaying interactions among authors in a research field. In this study, 100 authors with the most documents were chosen from a total of 7712 authors for author collaboration analysis. Figure 8 shows the 100 authors divided into 10 clusters, each of which might be considered a study group. For more information, see Table 5. This analysis is useful for future researchers who want to contact and interact with well-known and trending authors in the field of HFpEF research.

**Table 5:** Descriptive information about bibliographic networks.

**Bibliographic network**	**Cluster ID**	**N. Nodes**	**Representative nodes**
Author collaboration network	Cluster 1	16	Lam CSP; Vorrs AA; Van Veldhuisen DJ; Paulus WJ; De Boer RA
	Cluster 2	15	Borlaug BA; Redfield MM; Butler J; Hernandez AF; Fonarow GC
	Cluster 3	13	Shah SJ; Solomon SD; Pfeffer MA; Pitt B; Desai AS
	Cluster 4	12	Yamamoto K; Sakata Y; Chirinos JA; Saito Y; Yoshikawa T
	Cluster 5	11	Kitzman DW; Haykowsky MJ; Kaye DM; Brubaker PH; Pandey A
	Cluster 6	9	Zile MR; Mcmurray JJV; Anand IS; Komajda M; Carson PE
	Cluster 7	9	Pieske B; Edelmann F; Packer M; Hasenfuss G; Tschope C
	Cluster 8	7	Lund LH; Hage C; Donal E; Linde C; Daubert JC
	Cluster 9	6	Bonderman D; Mascherbauer J; Duca F; Aschauer S; Kammerlander AA
	Cluster 10	2	Nunez J; Palau P
Bibliographic coupling network	Cluster 1	34	Pitt B, 2014, New Engl J Med; Solomon SD, 2012, Lancet; Steinberg BA, 2012, Circulation; Berry C, 2012, Eur Heart J; Edelmann F, 2013, JAMA-J Am Med Assoc
	Cluster 2	32	Paulus WJ, 2013, J Am Coll Cardiol; Lam CSP, 2009, J Am Coll Cardiol; Shah SJ, 2016, Circulation; Mohammed SF, 2015, Circulation; Guazzi M, 2011, Circulation
	Cluster 3	18	Redfield MM, 2013, JAMA-J Am Med Assoc; Shah SJ, 2015, Circulation; Kraigher-Krainer E, 2014, J Am Coll Cardiol; Gonzalez-Lopez E, 2015, Eur Heart J; Zile MR, 2011, Circulation
	Cluster 4	16	Borlaug BA, 2011, Eur Heart J; Borlaug BA, 2010, Circ-Heart Fail; Borlaug BA, 2010, J Am Coll Cardiol; Kitzman DW, 2016, JAMA-J Am Med Assoc; Borlaug BA, 2014, Nat Rev Cardiol
References co-citation network	Cluster 1	35	Ponikowski P, 2016, Eur Heart J; Paulus WJ, 2013, J Am Coll Cardiol; Pitt B, 2014, New Engl J Med; Redfield MM, 2013, JAMA-J Am Med Assoc; Lam CSP, 2009, J Am Coll Cardiol
	Cluster 2	33	Owan TE, 2006, New Engl J Med; Massie BM, 2008, New Engl J Med; Yusuf S, 2003, Lancet; Bhatia RS, 2006, New Engl J Med; Cleland JGF, 2006, Eur Heart J
	Cluster 3	32	Paulus WJ, 2007, Eur Heart J; Borlaug BA, 2011, Eur Heart J; Borlaug BA, 2010, Circ-Heart Fail; Borlaug BA, 2010, J Am Coll Cardiol; Zile MR, 2004, New Engl J Med
Keyword co-occurrence network	Cluster 1	43	dysfunction; diastolic dysfunction; exercise capacity; diastolic function; hypertension
	Cluster 2	34	mortality; outcomes; association; prevalence; systolic function
	Cluster 3	23	diagnosis; echocardiography; guidelines; european-society; management

**Figure:**
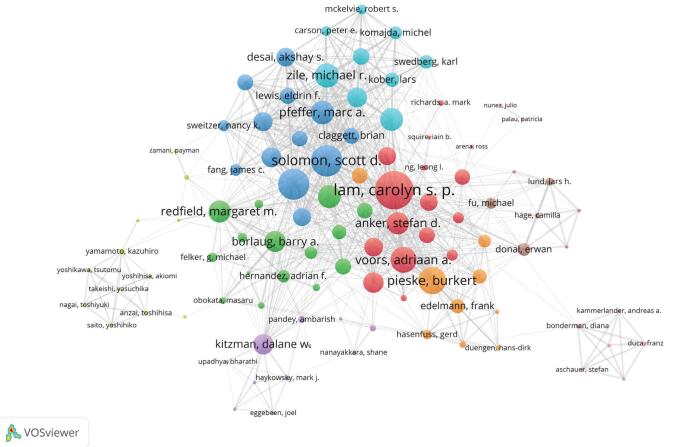
**Figure 8.** Author collaboration network. The color of a node is determined by the cluster to which the node belongs, and the size of the label and the circle of a node is proportional to the number of links of the node with other nodes.

Bibliographic coupling posits that 2 documents with public references have content that is similar.^[37]^ Based on shared references, the analysis builds a similarity link between documents and classifies them into clusters. The clusters are constructed based on the citing documents, which is worth highlighting. In this analysis, 100 documents from the local HFpEF literature collection with the greatest global citations were chosen for bibliographic coupling analysis. Figure 9 and Table 5 show the details of the 4 clusters that were created from the 100 documents.

**Figure:**
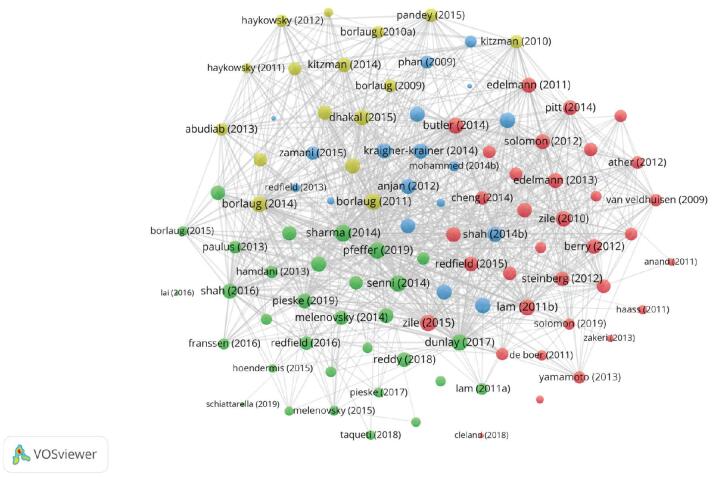
**Figure 9.** Bibliographic coupling network. The color of a node is determined by the cluster to which the node belongs, and the size of the label and the circle of a node is proportional to the number of links of the node with other nodes.

Another well-known bibliometric technique is co-citation analysis. This analysis assumes references that are cited together frequently are similar thematically.^[37]^ When 2 references appear in a document’s reference list at the same time, they are connected in a co-citation network. The most influential references and clusters can be discovered via co-citation analysis. It is worth noting that the clusters are derived based on the cited references. For reference co-citation analysis, 100 references most cited by the local documents were selected. Figure 10 and Table 5 demonstrate how the 100 references were divided into 3 clusters.

**Figure:**
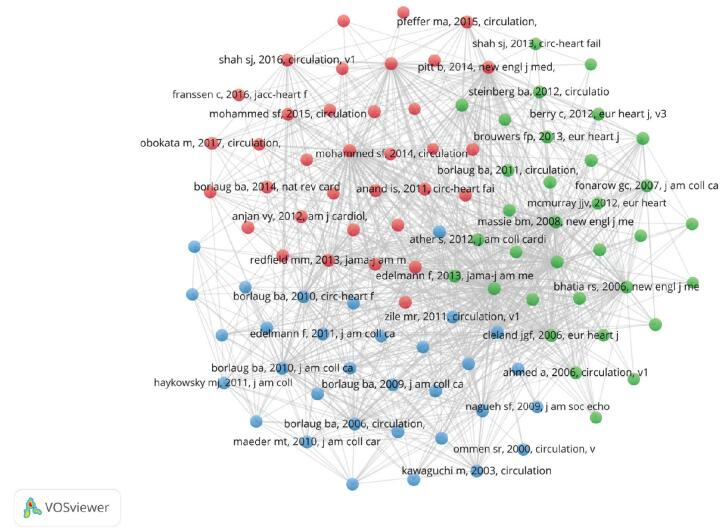
**Figure 10.** References co-citation network. The color of a node is determined by the cluster to which the node belongs, and the size of the label and the circle of a node is proportional to the number of links of the node with other nodes.

Keyword co-occurrence analysis is an approach that looks at the document’s real content.^[37]^ Keywords that commonly appear together are assumed to have a topic relationship in this analysis. Keyword co-occurrence analysis can reveal the study field’s development trends and hotspots. From the 169 terms listed above, 100 keywords with the most occurrences were chosen for co-occurrence analysis. As shown in Fig. 11 and Table 5, the 100 keywords were grouped into 3 clusters.

**Figure:**
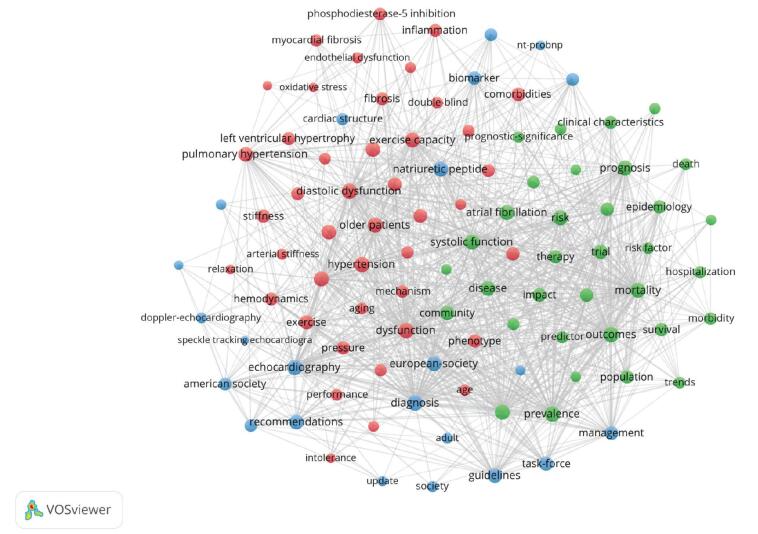
**Figure 11.** Keyword co-occurrence network. The color of a node is determined by the cluster to which the node belongs, and the size of the label and the circle of a node is proportional to the number of links of the node with other nodes.

## 4. Discussion

The pathogenesis of HFpEF is multifactorial.^[27]^ Because of the lack of a complete understanding of pathophysiology of HFpEF, modern heart failure medication has not improved the prognosis of HFpEF patients. The treatment of HFpEF patients is still a challenge for clinicians, and further research is urgently needed to evaluate new therapies to improve the treatment effect and longterm prognosis of HFpEF patients.

Based on the literature of HFpEF research from 2009 to 2020, this paper made a bibliometric analysis to fully understand the research trend of HFpEF in the past 12 years. Annual scientific production can reflect the development trend of specific research fields. HFpEF research had been developing steadily during the period, which indicated that HFpEF research attracted more and more attention and was in the growth stage.

Nine of the top-10 countries/regions with the highest productivity are developed countries/regions, including 2 in North America, 6 in Europe, and 1 in Asia. Seven of the top-10 affiliations of global HFpEF document output are in USA, 2 in Europe, and 1 in Asia. In addition, most of top-authors worked in USA and Europe. Developed countries/regions, especially USA, were particularly influential in the field of HFpEF research, while the majority of Asian, African, and Latin American countries/regions have relatively weak research capacity. There was an imbalance between countries/regions, and some research groups also lacked cooperation with the outside world. International cooperation that can pool the experience and wisdom of various countries/regions is an important way to accelerate scientific research progress and improve scientific research level. HFpEF research needs wider international cooperation.

All sources involved in Fig. 3B, D, F and Tables 2 and 3, except that the JCR category of New Engl J Med, JAMA-J Am Med Assoc, and Lancet is “Medicine, General & Internal,” the JCR category of other sources is “Cardiac & Cardiovascular Systems.” As shown in Table 6, New Engl J Med, JAMA-J Am Med Assoc and Lancet are all top journals with high reputation. Most “Cardiac & Cardiovascular Systems” sources, such as Eur Heart J, Circulation, Eur J Heart Fail, etc, also have high influence. Most HFpEF related documents were published in “Cardiac & Cardiovascular Systems” sources, especially those on heart failure. There were also some HFpEF related documents published in “Medicine, General & Internal” sources such as New Engl J Med, JAMA-J Am Med Assoc, and Lancet. Documents published in sources with high influence would receive high attention, and their citations were often high. HFpEF related documents published in these sources need to be focused by researchers interested in HFpEF.

**Table 6:** Some sources involved in HFpEF research field.

**JCR category**	**Source title**	**Latest impact factor**	**Category quartile**
Medicine, General & Internal	New Engl J Med	91.245	Q1
	Lancet	79.321	Q1
	JAMA-J Am Med Assoc	56.272	Q1
Cardiac & Cardiovascular Systems	Eur Heart J	29.983	Q1
	Circulation	29.690	Q1
	J Am Coll Cardiol	24.094	Q1
	Eur J Heart Fail	15.534	Q1
	JACC-Heart Fail	12.035	Q1
	Circ-Heart Fail	8.790	Q1
	J Card Fail	5.712	Q1
	J Am Heart Assoc	5.501	Q1
	Esc Heart Fail	4.411	Q2
	Int J Cardiol	4.164	Q2
	Am J Cardiol	2.778	Q3
HFpEF = heart failure with preserved ejection fraction.			

Compared to the reference Edlinger et al,^[45]^ this work reveals new research hotspots in the field of HFpEF in recent years through keyword analysis, one of which is phenotype. Phenotypes refer to identifiable patient groups, which are distinguished by both the presence of predominant presenting symptoms and/or predominant comorbidity profiles.^[23]^ The phenotypic heterogeneity of HFpEF is the main obstacle to HFpEF treatment.^[22]^ Phenotypic classification of HFpEF patients based on comorbidities and treatable etiologies can help determine the optimal management strategy for each individual patient.^[9]^ Therapeutics may need to shift from “one-size-fits-all” strategy to personalized approach based on phenotype characteristics.^[19,22,38,39]^ After diagnosing HFpEF, a potential approach is to use biomarkers to identify HFpEF phenotypes, guiding towards more appropriate therapeutic strategies.^[22]^ In addition, echocardiography can provide valuable information for further understanding of pathophysiology and potential phenotype of HFpEF.^[39]^ Machine learning have become important tool for biomedical knowledge discovery.^[40-43]^ Using machine learning to classify phenotypes of HFpEF patients without bias, high-quality evidence-based medical proofs may be produced, which can provide references for clinical decision-making. Oskouie et al^[44]^ has developed a method of machine learning to analyze phenotypes, which divides HFpEF patients into 3 phenotype groups with different clinical characteristics and results. Segar et al^[20]^ also used unsupervised clustering analysis method to classify HFpEF patients in high-dimensional mixed data queue. The results showed that cluster analysis based on machine learning can identify phenotype groups of HFpEF patients with different clinical characteristics. Using biomarkers from HFpEF patients for unsupervised cluster analysis, Woolley et al^[25]^ identified 4 mutually exclusive patient subgroups according to the characteristics of biomarkers, which suggested different underlying pathophysiological pathways. In view of the phenotypic heterogeneity of HFpEF, treatment and clinical research need to be individualized and targeted. Researchers should abandon the mindset of “a pair of shoes for all feet,” and use machine learning algorithm to divide HFpEF patients into different phenotype groups, which can make the patients better match the appropriate treatment and improve the prognosis.^[21]^ Using machine learning to classify the phenotypes of HFpEF patients is a very noteworthy research hotspot.

Despite an exhaustive bibliometric analysis of HFpEF study, deficiencies remain. Due to the time limit of WoS subscribed by our institution, this study could only query the literature after 2009, which might lead to the incompleteness of the local HFpEF literature collection. In addition, this study took title as the query item to search HFpEF literature, which improved the precision, but might reduce the recall. We have tried to search for HFpEF-related papers using keywords, but the search results are not satisfactory. Many of the documents are not very relevant to HFpEF research and may only cover a small part of the content. We believe that HFpEF-related document retrieval by title will maximize the inclusion of the most important documents. For the annual analysis of HFpEF literature, the local HFpEF literature collection did not contain the latest HFpEF documents since 2021. In August 2021, however, an important breakthrough was reported. The sodium-glucose cotransporter 2 inhibitors were shown to be effective in the treatment of HFpEF.^[46,47]^ This is another research hotspot worthy of attention. Furthermore, this study only processed the bibliographic database files from WoS, and did not consider other bibliographic databases, such as PubMed, Scopus, etc. These above deficiencies did not undermine the significance of this study.

## 5. Conclusion

With the sharp increase in the number of HFpEF patients, HFpEF is recognized as a major public health problem worldwide. Through bibliometric analysis of HFpEF literature from 2009 to 2020, this study provided a comprehensive reference and a summarized macroscopic overview of the research progress and performance related to HFpEF.

The bibliometric analytic tools employed in this study gleaned relevant information from published documents, supplemented standard review and survey articles, and allowed researchers to gain useful information. Over time, there has been an increase in the amount of HFpEF documents, however HFpEF research has been relatively imbalanced amongst countries/regions. It is necessary to conduct more cooperative and collective global HFpEF research in order to conduct HFpEF research in a balanced manner around the world. This study aids HFpEF researchers in locating possible collaborators and identifying research trends and hotspots, all of which will assist improve the treatment and prognosis of HFpEF patients around the world.

## Acknowledgments

The authors are particularly grateful to Zhenyu Feng, Qinglian Hao, and Tao Lin.

## Author contributions

**Conceptualization:** Qiuju Dong, Junwei Zhang.

**Data curation:** Qiuju Dong, Junwei Zhang.

**Funding acquisition:** Qiuju Dong, Jianping Zhao.

**Investigation:** Hongzhen Zhang, Meiling Wang.

**Methodology:** Qiuju Dong, Junwei Zhang.

**Project administration:** Jianping Zhao.

**Supervision:** Qinghua Han.

**Visualization:** Junwei Zhang.

**Writing** - **original draft:** Qiuju Dong, Junwei Zhang, Qi Huang.
